# Suggestions Made by a Committee of Veterinarians to the Mayor of Philadelphia Relative to the Meat-Inspection Service

**Published:** 1899-10

**Authors:** 


### SUGGESTIONS MADE BY A COMMITTEE OF VETERINARIANS TO THE MAYOR OF PHILADELPHIA RELATIVE TO THE MEAT-INSPECTION SERVICE.

THE meat-inspection work of the city of Philadelphia is at present performed by a chief meat-detective, an associate meat-detective, a veterinarian for meat-inspection, and a consulting veterinarian for meat-inspection. These officials constitute the entire meat-inspection force of Philadelphia, with the exception of the market clerks, who have a little incidental control over the meat sold in their respective markets.

The business of slaughtering in Philadelphia is scattered enormously. Food-animals are killed in all parts of the city, in small, isolated slaughter-houses, and at irregular and indefinite times. The natural result is that only a small percentage of the animals killed for food are seen by the inspectors. The inspectors make regular visits to a few slaughter-houses. These visits are, necessarily, made at long intervals and for brief periods. If a diseased animal has just been killed, and is being dressed, it is likely to be detected by the inspector and condemned. Many of the butchers are wary and unscrupulous, and destroy and conceal evidence of disease found in animals killed during the absence of the inspector. If an animal is suspected of being in a condition to lead to its condemnation, it is killed at a time when the inspector is not apt to be present, as before or after regular business hours, and sometimes at midnight.

The inspectors are energetic men, who are doing as much to pro-

[1] Archiv. f. Exp. Path. u. Pharmak., Bd. xxvii, p. 432.
[2] Deutsche med. Wochenschrift, December 4, 1890, No. 49, p. 1113.

tect the public as is possible under existing conditions. It is quite evident, however, that they cannot be expected to detect more than a small percentage of the diseased meat prepared in Philadelphia. They have, however, succeeded in making butchers cautious, and the business of slaughtering diseased animals is not carried on so glaringly as it was before the present meat-inspection was established.

What is needed is a system that will make it possible to inspect every meat-producing animal, particularly every beef-animal, before and after slaughter. If this cannot be accomplished at present there is immediate, urgent need of a system that will make it possible to inspect the organs of every beef-animal before the meat goes on to the market. Such a system could be arranged without material change in the existing conditions under which the slaughtering business is carried on, and without expensive additions to the meat-inspection force. As a means to this end the following recommendations are made:

1. All meat inspected by the meat-inspector should be stamped.
2. The sale of unstamped meat should be prohibited.
3. The hours for slaughtering should be regulated and butchers should be prohibited from killing animals at times other than those fixed.

This regulation need involve no hardship to the butcher. The hours established for the different slaughter-houses could vary according to the customs of the butchers, but the hours once fixed should be strenuously adhered to until changed by agreement between the chief meat-inspector and the butcher, and upon a written request of the butcher. If the exigencies of business should make it impossible for the butcher to supply his trade without killing at a time outside of the period arranged, it could be provided that temporary permission to slaughter at such additional time should be had after twelve hours' notice to the chief meat-inspector.

4. All slaughter-houses should be visited by the meat-inspectors during the hours of slaughtering, and all of the carcasses should be examined, and those found to be in a condition suitable for food should be stamped.

The butcher should be required to keep certain of the organs, especially the lungs and liver, attached to the carcass until after it has been examined by the meat-inspector.

5. Western beef should be examined and stamped if it is found to be in good condition, and to bear the inspection tag of the Federal inspector on duty in the slaughter-house in which it is prepared.

6. Beef prepared in the surrounding country and brought into the city in wagons should be brought in only at certain specified times and before unloading the wagon should pass certain inspection points where the meat could be examined by an inspector stationed for this purpose, and stamped if found to be in sound condition.

The lungs and liver should be attached to each carcass brought into the city from the surrounding country.

7. The meat-inspection force should be increased by the addition of at least two veterinarians.

Meat-inspection is within the domain of veterinary science, and is taught only in veterinary colleges. The meat-inspectors of other countries and those employed by the Federal Government must be veterinarians. Meat-inspection is applied comparative pathology, and veterinarians are the only men regularly trained in the pathology of domestic animals.

8. Sanitary regulations should be established governing certain features of the construction, fittings, and care of slaughter-houses, and all slaughter-houses falling below a reasonable standard should be proceeded against as nuisances.

At present many of the slaughter-houses are of the most filthy and abominable description, without space or facilities for the preparation of wholesome meat. Some are without suitable drainage, are kept in a nasty condition, and the most disgusting practices are carried on in them.

---

Inflating the Udder. An experienced Scotch herdsman explains that inflation of the cow's udder was practised more or less in the "old country" by professional herdsmen and showmen, but he did not suppose the practice was resorted to in any other country. He went on to say that in Great Britain it had been found that injecting into the udder new milk which had been carefully drawn, and was absolutely pure and of the same temperature as when drawn from the udder, had the effect of distending the udder considerably, and if properly done and no air admitted to the udder during the operation it was not likely to prove a permanent injury to the cow, while, on the other hand, if skimmed milk were used, even though it were of the desired temperature, bad results were likely to follow, and the same might be expected if water was used, though pure distilled water would, perhaps, not cause death. The inflation of the udder with air he believed to be fatal in every case.—*Jersey Bulletin.*

